# Long non-coding RNAs in biomarking COVID-19: a machine learning-based approach

**DOI:** 10.1186/s12985-024-02408-9

**Published:** 2024-06-07

**Authors:** Raheleh Heydari, Mohammad Javad Tavassolifar, Sara Fayazzadeh, Omid Sadatpour, Anna Meyfour

**Affiliations:** 1 Basic and Molecular Epidemiology of Gastrointestinal Disorders Research Center, Research Institute for Gastroenterology and Liver Diseases, Shahid Beheshti University of Medical Sciences, Tehran, Iran; 2 Bioinformatics and Computational Omics Lab (BioCOOL), Department of Biophysics, Faculty of Biological Sciences, Tarbiat Modares University, Tehran, Iran; 3 Department of Immunology, School of Medicine, Tehran University of Medical Sciences, Tehran, Iran

**Keywords:** COVID-19, Long non-coding RNA, Peripheral blood mononuclear cell, Diagnosis, Machine learning

## Abstract

**Background:**

The coronavirus pandemic that started in 2019 has caused the highest mortality and morbidity rates worldwide. Data on the role of long non-coding RNAs (lncRNAs) in coronavirus disease 2019 (COVID-19) is scarce. We aimed to elucidate the relationship of three important lncRNAs in the inflammatory states, H19, taurine upregulated gene 1 (TUG1), and colorectal neoplasia differentially expressed (CRNDE) with key factors in inflammation and fibrosis induction including signal transducer and activator of transcription3 (STAT3), alpha smooth muscle actin (α-SMA), tumor necrosis factor-alpha (TNF-α), and interleukin-6 (IL-6) in COVID-19 patients with moderate to severe symptoms.

**Methods:**

Peripheral blood mononuclear cells from 28 COVID-19 patients and 17 healthy controls were collected. The real-time quantitative polymerase chain reaction (RT-qPCR) was performed to evaluate the expression of RNAs and lncRNAs. Western blotting analysis was also performed to determine the expression levels of STAT3 and α-SMA proteins. Machine learning and receiver operating characteristic (ROC) curve analysis were carried out to evaluate the distinguishing ability of lncRNAs.

**Results:**

The expression levels of H19, TUG1, and CRNDE were significantly overexpressed in COVID-19 patients compared to healthy controls. Moreover, STAT3 and α-SMA expression levels were remarkedly increased at both transcript and protein levels in patients with COVID-19 compared to healthy subjects and were correlated with Three lncRNAs. Likewise, IL-6 and TNF-α were considerably upregulated in COVID-19 patients. Machine learning and ROC curve analysis showed that CRNDE-H19 panel has the proper ability to distinguish COVID-19 patients from healthy individuals (area under the curve (AUC) = 0.86).

**Conclusion:**

The overexpression of three lncRNAs in COVID-19 patients observed in this study may align with significant manifestations of COVID-19. Furthermore, their co-expression with STAT3 and α-SMA, two critical factors implicated in inflammation and fibrosis induction, underscores their potential involvement in exacerbating cardiovascular, pulmonary and common symptoms and complications associated with COVID-19. The combination of CRNDE and H19 lncRNAs seems to be an impressive host-based biomarker panel for screening and diagnosis of COVID-19 patients from healthy controls. Research into lncRNAs can provide a robust platform to find new viral infection-related mediators and propose novel therapeutic strategies for viral infections and immune disorders.

**Supplementary Information:**

The online version contains supplementary material available at 10.1186/s12985-024-02408-9.

## Introduction

Due to the accelerated geographic expansion of coronavirus over the past two decades, it has become a real hazard to the health of the world. A respiratory sickness called Coronavirus Disease 2019 (COVID-19) is caused by the “severe acute respiratory syndrome coronavirus 2” (SARS-CoV-2), which is a single-stranded, positive-sense RNA virus with an envelope that belongs to the Betacoronavirus genus, Coronaviridae family [1, 2]. More than 774 million confirmed infections and over 7 million deaths have been globally reported until 16 February 2024 [3] SARS-CoV-2 enters the host cells using ACE2 receptor which is expressed in many tissues, including upper respiratory airways, as the primary entry site of the virus. This widespread expression of the ACE2 receptor explains the multisystem presentations of this infection [4]. Recently, the association of expression level of ACE2 variants with various contributing factors related to COVID-19 severity has been reviewed. These factors include age, gender, and comorbidities such as cardiovascular diseases, chronic respiratory diseases, diabetes, and obesity Significantly higher expression levels of ACE2 were observed in more severe cases of COVID-19, suggesting that an increase in SARS2-CoV-2 receptors may contribute to the heightened severity of the disease [5–7] The majority of COVID-19 patients experience minor health issues; however, these can worsen, particularly in the elderly or in those with underlying conditions such as chronic lung or cardiovascular diseases. The emergence of the immune-mediated COVID-19 cytokine storm (CCS) is one of the most remarkable clinical symptoms of the pandemic. The clinical condition appears toward the end of the first week after infection, followed by a severe catastrophic hyper-immune pro-inflammatory reaction which is marked by a high-level production of pro-inflammatory cytokines such as tumor necrosis factor-alpha (TNF-α), interleukin-6 (IL-6), and interleukin-8 (IL-8) [4]. The possible multisystem symptoms of this hyper-immune pro-inflammatory response include the substantial vascular permeability that results in secondary cardiovascular problems, a hypercoagulable condition that causes tissue inflammation, damage, and severe amounts of venous and arterial thromboembolism leading to acute respiratory distress syndrome (ARDS) which is the main cause of these patients’ deaths [4, 8]. It has been observed that various types of RNAs, including microRNAs (miRNAs), cell-free RNAs (cfRNAa), and long noncoding RNAs (lncRNAs), are correlated with various inflammatory diseases and infectious diseases, including COVID-19 [9–12], providing insight into potential mechanisms involved in the pathogenesis of these diseases. Therefore, they could serve as valuable prognostic biomarkers. Jalaleddine et al. identified a significant relationship between COVID-19 severity and ACE2-cfRNA levels in liquid biopsies from COVID-19 patients. They suggested monitoring ACE2-cfRNAs as a potential biomarker during COVID-19 infection [13]. Another recent study reported a comparative analysis of plasma cfRNA profiles between COVID-19 patients and healthy subjects. They found that lncRNA GJA9-MYCBP was upregulated in severe COVID-19 patients compared to healthy subjects. This lncRNA carries a binding site for certain miRNAs from the let-7 family, which in turn increases the protein expression levels of IL6 and IL6R, thereby promoting cytokine storm syndromes in COVID-19 patients [11].

RNA transcripts with more than 200 nucleotides which do not code for proteins are called lncRNAs. These RNAs lack open reading frames (ORF) that are necessary for protein coding. LncRNAs are expressed in most tissues and play critical roles in various cellular processes, including gene regulation, chromatin remodeling, cell cycle, differentiation, and metabolism under physiological and pathophysiological conditions [14–16].

One of the aspects affected by lncRNAs is cytokine production, which is of great importance in COVID-19 patients, as cytokine storm and the probable ARDS state are observed in severe cases of the disease [17, 18]. To date, lots of lncRNAs have been known to be involved in the pathogenesis of different diseases and even recent studies have provided evidence for their role in COVID-19 [18–20]. Taurine upregulated gene 1 (TUG1), also known as TI-227 H, LINC00080, and NCRNA00080, is located on human chromosome 22 (22 q12.2) and has about 7.1 kb. This lncRNA was first recognized as being involved in mouse retinal cells development [21]. Accumulating data have demonstrated TUG1 dysregulation in various pathological condition such as osteosarcoma, obesity, bladder cancer, and gastric cancer (GC) as well as inflammatory diseases and infections; therefore, it has been introduced as a therapeutic target in different diseases [21–23]. Lv et al. demonstrated that increased expression level of lncRNA TUG1 interacting with miR-144 promoted proliferation and migration of in hepatocellular carcinoma (HCC) cells via activating the Janus kinase 2 (JAK2)/ signal transducer and activator of transcription3 (STAT3) signaling pathway [24]. Additionally, lncRNAs TUG1 has been shown significantly upregulation in lung tissues of chronic obstructive pulmonary disease (COPD) patients compared to non-COPD lung tissues and its silencing enhanced proliferation of transforming growth factor β (TGF-β) induced human lung epithelial cells (BEAS-2B) and human lung fibroblasts (HFL1) cells through preventing the expression levels of alpha smooth muscle actin (α-SMA) and fibronectin [25].

H19 is another lncRNA whose aberrant expression has been observed in some pathophysiological and inflammatory states. This lncRNA belongs to a conserved gene cluster located on chromosome 11 (11p15.5) that produces a 2.3 kb spliced, polyadenylated and capped lncRNA [17]. During embryogenesis, H19 expression is boosted, but it rapidly declines after delivery. However, its expression in the skeletal muscle and cartilage persists even after birth [26]. H19 expression is elevated in coronary artery disease (CAD) [27]. Although the exact mechanism of its involvement in CAD is still unknown, it could be related to the significant H19 role in atherosclerosis which is a very important phase in CAD development [27, 28]. Furthermore, H19 contribution to many other inflammatory conditions, such as nephrotic syndrome, type 2 diabetes, and liver diseases, has been observed, highlighting its modulatory effects on inflammatory responses [29–31]. Furthermore, overexpression of lncRNA H19 has been shown to enhance epithelial-mesenchymal transition (EMT) and metastasis through let7c/STAT3/enhancer Of Zeste 2 Polycomb Repressive Complex 2 Subunit (EZH2)/β-catenin pathway in esophageal cancer (EC). The upregulation of H19 significantly increased the mRNA and protein levels of STAT3, EZH2 and β-catenin, suggesting that STAT3 is regulated by H19 in EC [32].

LncRNA colorectal neoplasia differentially expressed (CRNDE) is located on chromosome 16 whose association with colorectal cancer has been shown in previous reports [33]. Upregulation of CRNDE has been found as one of the important causes of cancer cell proliferation and metastasis while CRNDE knockdown could induce apoptosis in cancer cells [33]. Furthermore, overexpression of CRNDE has shown a close connection with inflammation caused by LPS, alcoholic liver disease, and sepsis [34–36]. Zhu-ge et al. discovered that lncRNA CRNDE overexpression increased cell injuries, cell apoptosis and inflammatory cytokines levels in LPS-injured human lung fibroblast cell line (WI-38 cells). In addition, upregulation of CRNDE increased the activity of Jak1, pTyk2 and Stat3 in the NF-κB and JAK/STAT signaling pathways [36]. Considering the important roles of lncRNAs in a vast spectrum of inflammatory diseases and infections as well as lack of sufficient data regarding the involvement of lncRNAs in COVID-19 pathogenesis, we aimed to assess and compare the expression level of three of the most important lncRNAs H19, TUG1, and CRNDE in the peripheral blood mononuclear cells (PBMCs) of moderate to severe COVID-19 patients and healthy controls.

Furthermore, their associations with four key players in the inflammation and fibrosis including STAT3 [37], α-SMA [38], IL-6, and TNF-α [39, 40] were evaluated to highlight their possible contribution to exacerbation of symptoms and COVID-19 complications in cardiovascular, respiratory, and other systems.

## Materials and methods

### Patients

Whole blood (WB) specimens were obtained from 28 patients with moderate to severe SARS-CoV-2 infection confirmed using clinical symptoms and real-time quantitative polymerase chain reaction (RT-qPCR) testing on nasal (NP) and oropharyngeal (OP) swabs as the gold standard test to confirm SARS-CoV-2 infection. Furthermore, whole blood (WB) samples from 17 healthy sex-and age-matched volunteers with a negative RT-qPCR result of SARS-CoV-2 infection and no history of COVID-19 were obtained in Taleghani hospital. Participants had to meet the following requirements in order to be included: age of 18 or older, no disease-modifying therapies (DMTs) for COVID-19, diagnosis of SARS-CoV-2 infection using RT-qPCR from nasal and oropharyngeal swabs. Moreover, the patients with COVID-19 and donor controls had not taken any antioxidant supplements for at least four weeks prior to sampling. The study process was explained to the participants, and their consent was gained. The ethics committee approved the study of Shahid Beheshti University of Medical Sciences (SBMU) (Reference No.IR.SBMU.RIGLD.REC.1403.022).

### PBMC isolation

The WB samples were collected in EDTA-coated tubes (10 ml each) from the clinical laboratory of Taleghani hospital for the investigation. Ficoll-Hypaque density gradient medium (Lymphodex, inno-train, Germany) in a ratio of 2:1 (2 blood:1 Ficoll) was used to isolate PBMCs. Then, PBMCs were centrifuged at 1500 rpm for 15 min at 20 °C and washed twice with phosphate-buffered saline (PBS). Finally, the viability of isolated PBMCs was assessed by trypan blue staining and observed by light microscopy (Nikon E100 Binocular Microscope, Japan). The viability of isolated PBMCs was evaluated to be more than 98%.

### RT-qPCR

Total RNA was extracted from PBMC samples of COVID-19 patients and healthy controls using the TRIzol-LS reagent (Ambion, Life Technology, USA), according to the manufacturer’s instructions to evaluate the expression levels of H19, CRNDE, TUG1, STAT3, α-SMA, IL_6, and TNF-α. RNA concentration and integrity were assessed by NanoDrop (Thermo Fisher) and gel analysis. Subsequently, the isolated total RNA was reverse transcribed to complementary DNA (cDNA) using cDNA synthesis kit (Parstous, Iran). Then, RT-qPCR was performed using 2x SYBR Green qPCR Mix plus (Ampliqone, Denmark) on a Rotor-Gene Q System (Qiagen). The relative expression levels of genes were evaluated and normalized by the 2^− ΔΔ CT^ using glyceraldehyde 3-phosphate dehydrogenase (GAPDH) as an internal control. The sequences of primers are listed in Table 1.

**Table 1:** The oligonucleotide sequences of presented primers

Primer name	Primers sequences
TUG1-Forward TUG1-Reverse	ACCGGAGGAGCCATCTTGTC GAAAGAGCCGCCAACCGATC
CRNDE-Forward CRNDE-Reverse	GTTGTCACGCAGAAGAAG TCCTATACCTTGGCTAAACA
H19-Forward H19-Reverse	GGGTCTGTTTCTTTACTT TAGCACCATTTCTTTCAT
α-SMA-Forward α-SMA-Reverse	GGCACCACTGAACCCTAAG AATACCAGTTGTACGTCCAGA
STAT3-Forward STAT3-Reverse	CAAAGAAAACATGGCTGGCA TGAAACCCATGATGTACCCTT
IL-6-Forward IL-6-Reverse	ACTCACCTCTTCAGAACGAATTG CCATCTTTGGAAGGTTCAGGTTG
TNF-α-Forward TNF-α-Reverse	CCATGTTGTAGCAAACCCT GGACCTGGGAGTAGATGAG
GAPDH-Forward GAPDH-Reverse	CTCATTTCCTGGTATGACAACGA CTTCCTCTTGTGCTCTTGCT

### Western blot analysis to determine protein level

Total proteins were extracted from PBMCs using the TRIzol-LS reagent (Ambion, Life Technology, USA), according to the manufacturer’s protocol. Protein concentration was determined using bicinchoninic acid assay (BCA) kit (Thermo Fisher Scientific). Almost 25 μg protein was loaded onto 12% sodium dodecyl sulfate polyacrylamide electrophoresis (SDS-PAGE) gel for separation. Resolved proteins were then transferred from gels to polyvinylidene difluoride (PVDF) membrane. The membranes were blocked with 5% non-fat milk for 2 h (h) at room temperature (RT), incubated with primary antibodies, including anti-α-SMA (1:1000; Invitrogen) and anti-STAT3 (0.1 μg/mL, R&D) at 4 °C overnight. Then, membranes were washed three times for 10 min in Tris-Buffered Saline with Tween-20 (TBST), and incubated with secondary antibody (Thermo Fisher Scientific) for 1 h at RT. This was followed by three times washing for 10 min in TBST and incubation in ECL substrate chemiluminescent detection reagent (Pierce ™ ECL Western Blotting Substrate, Thermo Fisher Scientific). Protein bands were then visualized by imaging system (Fusion solo S, Vilber lourmat). β‐Actin was also used as a loading control.

### In-silico expression validation

The Gene Expression Omnibus (GEO) database (https://www.ncbi.nlm.nih.gov/geo/) was queried to identify datasets focusing on the evaluation of lncRNAs in PBMCs of COVID-19 patients. A dataset containing the lncRNA expression profiles of PBMCs from five severe COVID-19 patients and five healthy individuals was identified (GSE164805). We conducted a differential expression analysis to statistically compare severe COVID-19 patients with healthy controls. The lncRNAs with an absolute log_2_(fold change) greater than 1 and adjusted *P-value* less than 0.05 were deemed statistically differentially expressed. For this purpose, we utilized the limma package in R. We assessed whether the literature-based candidates were present among the differentially expressed lncRNAs in the retrieved dataset. Expression barplots of the candidate lncRNAs were generated using GraphPad Prism.

### Machine learning

To predict COVID-19 status in comparison to healthy controls, the logistic regression (LR) algorithm was employed. LR has been extensively applied over the last two decades in the medical and health sciences fields and is considered a standard approach for constructing predictive models in binary classification problems, particularly in research involving disease states (diseased/healthy) and decision-making processes. This algorithm calculates the probability that a given sample belongs to a specific class. It elucidates the relationship between independent variables and a dependent variable, with the coefficients in an LR model indicating the degree of association between them. Utilizing this method, LR models were constructed and validated for individual lncRNAs and their combinations using 5-fold cross-validation in R. The performance of the models was evaluated based on sensitivity, specificity, and accuracy. Furthermore, the area under the receiver operating characteristic (ROC) curve (AUC) was calculated for each model as an independent assessment metric.

### Statistical analysis

The expression levels of lncRNAs and genes in COVID-19 patients and healthy controls were compared by the t-test. Statistical analysis and graph construction were performed using Prism (GraphPad software Inc). Data are expressed as mean ± SEM. Results with *P-value* < 0.05 were considered statistically significant.

Furthermore, we performed a correlation analysis between the lncRNAs (TUG1, CRNDE, and H19) and genes (STAT3, α-SMA, TNF-α, and IL-6) and visualized the plot using the corrplot package in R.

## Results

### Basic clinical characteristics and patients’ information

In this study, 28 COVID-19 patients and 17 healthy controls were age and gender-matched. The mean age ± SD of the patients and control subjects was 54.68 ± 17.19and 51.15 ± 17.73, respectively. Males represented 46.43% of the patient group and 53% of the control group. The mean ± SD oxygen saturation of COVID-19 patients and healthy controls were 84.11 ± 5.93 and 94.45 ± 0.66, respectively. In addition, the mean WBC and platelet counts of COVID-19 patients were 6.87 ± 3.44 (10^3^/μL) and 142.47 ± 79.55 (10^3^/μL), respectively. Other clinical and laboratory parameters of COVID-19 patients are listed in Tables 2 and 3, respectively.

**Table 2:** Demographic characteristics of the COVID-19 patients and control groups

Factors	COVID-19 Group *N* = 28	Control Group *N* = 17
Age	54.68 ± 17.19	51.15 ± 17.73
Sex		
Male	13 (46.43)	9 (53)
Female	15 (53.57)	8 (47)
Smoking		
Yes	11 (39.3)	7 (41.2)
No	17 (60.7)	10 (58.8)
Comorbidity		
Yes	15 (53.57)	2 (11.8)
No	13 (46.43)	15 (88.2)
Comorbidity		
DM	9 (30)	0 (0.0)
HTN	10 (33)	2 (10)
Hepatic	4 (13)	0 (0.0)
Renal	3 (10)	0 (0.0)
Cardiac	5 (17)	0 (0.0)
Tachycardia	9 (30)	0 (0.0)
Fever	16 (53)	0 (0.0)
Dyspnea	12 (40)	0 (0.0)
Cough	21 (70)	0 (0.0)
Wheezy	10 (33)	0 (0.0)
Diarrhea	5 (17)	0 (0.0)
Outcome		
Survived	24 (85.7)	
Died	4 (14.3)	

Diabetes (DM); hypertension (HTN)

**Table 3:** Laboratory parameters of the COVID-19 patients and control groups

Clinical factors	COVID-19 Group *N* = 28	Control Group *N* = 17
Oxygen concentration (%)	84.11 ± 5.93	94.45 ± 0.66
WBC (10^3^/μL)	6.87 ± 3.44	6.70 ± 1.67
Hb (g/dL)	10.66 ± 1.60	14.27 ± 0.98
PLT (10^3^/μL)	142.47 ± 79.55	264.55 ± 78.02
FBS (mg/dL)	144.61 ± 42.63	92.93 ± 5.28
BUN (mg/dL)	17.55 ± 10.73	17.56 ± 8.20
Creatinine (mg/dL)	1.43 ± 1.18	0.74 ± 0.09
Na (mEq/L)	139.83 ± 2.99	140.5 ± 2.37
K (mEq/L)	4.53 ± 0.71	4.05 ± 0.32
LDH (U/L)	695.82 ± 354.95	213.86 ± 49.38
AST (U/L)	47.2 ± 29.92	16.95 ± 4.04
ALT (U/L)	43 ± 27.50	13.05 ± 3.23
CRP (mg/dL)	77.22 ± 41.57	3.23 ± 1.24
ESR (mm/hour)	47.68 ± 26.65	9.25 ± 5.37

### Increased expression of H19, TUG1, and CRNDE in COVID-19 patients

**Fig. 1:**
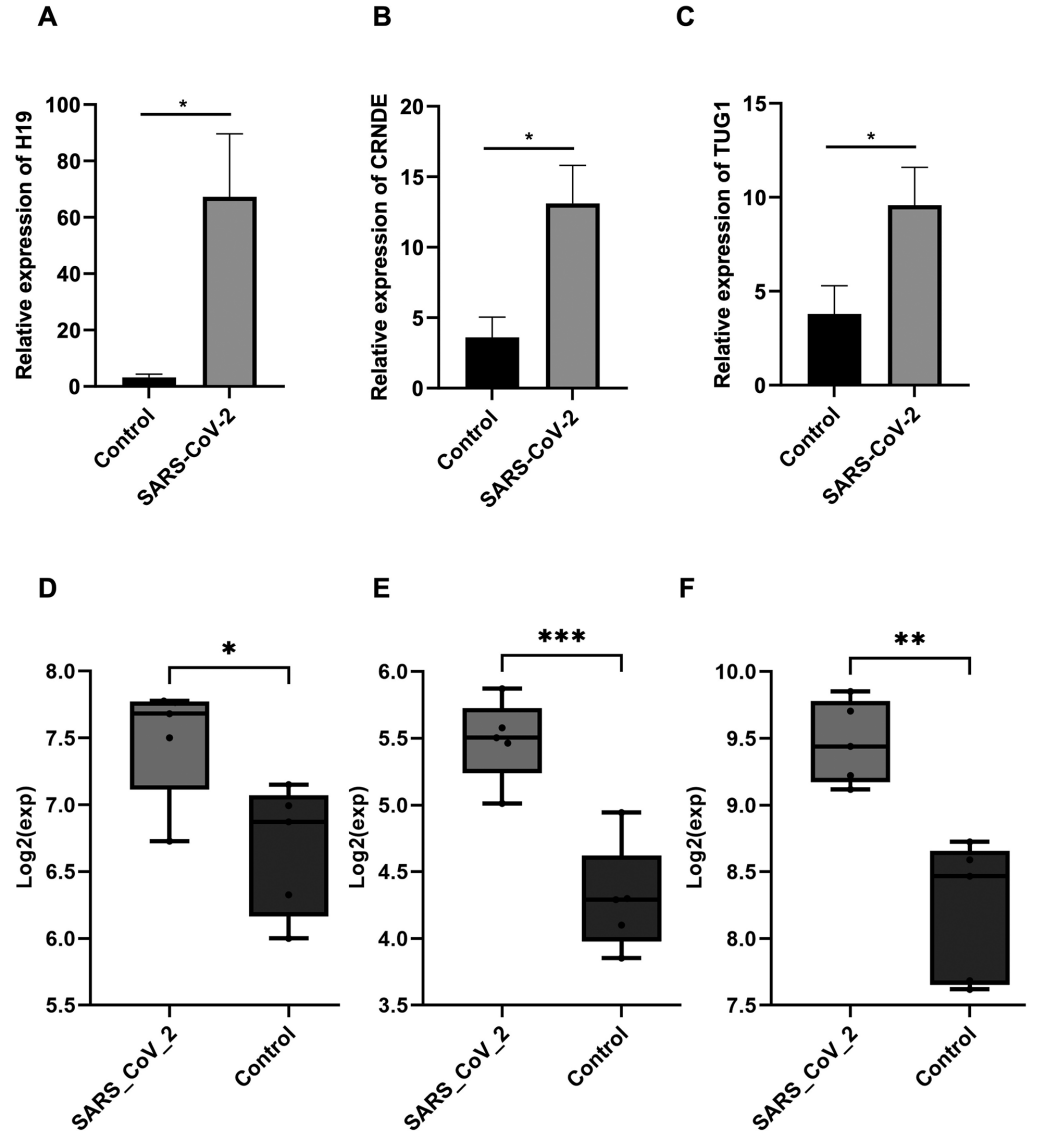
Dysregulation of lncRNAs in PBMCs of COVID-19 patients. Expression of lncRNA (**A**) H19; (**B**) CRNDE; and (**C**) TUG1 was increased in COVID-19 patients compared to healthy individuals. The gene expression data were normalized to GAPDH. Data are expressed as mean ± SEM. Validation of (**D**) TUG1, (**E**) CRNDE, and (**F**) H19 expression changes in the in-silico dataset obtained from GEO database ((*P-value < 0.05, **P-value < 0.01; ***P-value < 0.001)

To investigate the role of lncRNAs in COVID-19, we analyzed changes in the expression of three significant lncRNAs, known for their crucial roles in various inflammatory states, in the PBMC samples of COVID-19 patients. Intriguingly, the expression levels of H19, TUG1, and CRNDE were significantly increased in COVID-19 patients compared to the healthy controls (Fig. 1A-C). These results suggest that SARS-CoV-2 influences the expression of these lncRNAs.

We also conducted a differential expression analysis to assess the expression of these lncRNAs in an *in-silico* dataset on the PBMC samples from COVID-19 patients and healthy controls. We observed that all three lncRNAs were significantly upregulated in COVID-19 patients of the dataset obtained from GEO (Fig. 1D-F), and these findings were consistent with the results obtained from our analysis.

### Increased expression of genes involved in inflammation and fibrosis induction in COVID-19 patients

**Fig. 2:**
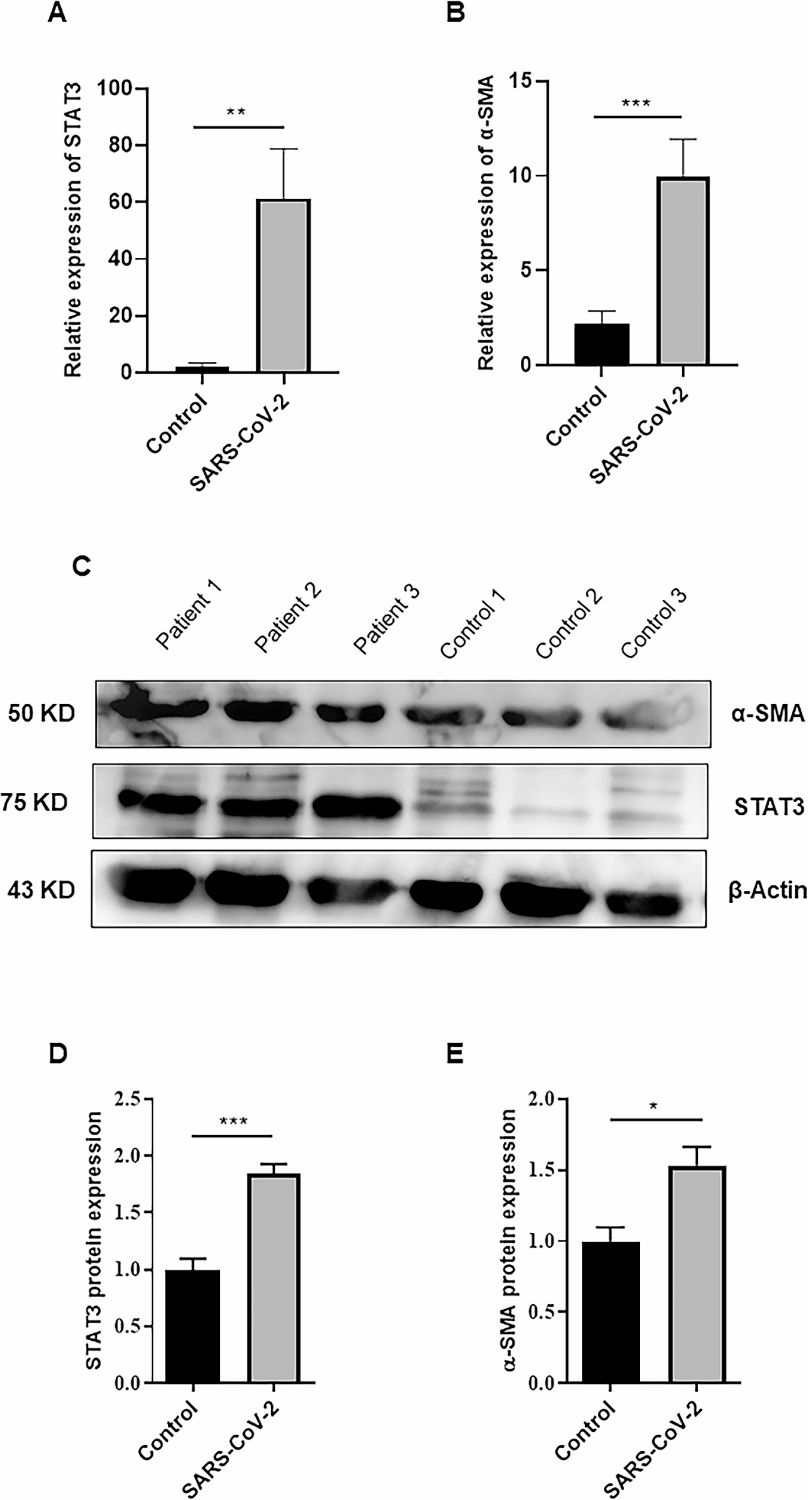
Changes in the expression of STAT3 and α-SMA in PBMCs of COVID-19 patients. The expression of (**A**) STAT3 and (**B**) α-SMA transcripts. The gene expression data were normalized to GAPDH. (**C**) The protein expression levels of STAT3 and α-SMA were investigated in PBMCs of COVID-19 patients compared to healthy controls. The histogram represented the intensity of (**D**) STAT3 and (**E**) α-SMA proteins for each group which was normalized to the β-actin band; **P-value < 0.01; ***P-value < 0.001

To better understand the mechanisms through which the studied lncRNAs might have an impact, we decided to assess the expression levels of STAT3 and α-SMA transcripts. Several studies have demonstrated that these lncRNAs have downstream targets and exert their effect through dysregulation of STAT3 and α-SMA [25, 41, 42]. We observed increased expression of STAT3 and α-SMA in COVID-19 patients compared to healthy controls (Fig. 2A-B). Furthermore, the protein levels of STAT3 and α-SMA were evaluated in COVID-19 patients which showed overexpression patterns compared to the control group (Fig. 2C-E, and Supplementary Fig. 1), similar to what was observed at their transcript levels.

**Fig. 3:**
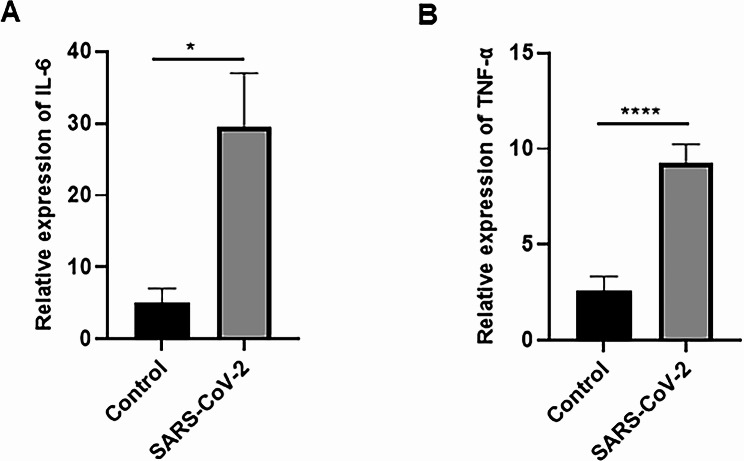
Dysregulation of pro-inflammatory cytokines in PBMCs of COVID-19 patients. (**A**) IL-6 and (**B**) TNF-α were overexpressed in patients with COVID-19 compared to healthy subjects. The gene expression data were normalized to GAPDH. Data for gene expression levels are presented as mean ± SEM (*P-value < 0.05; ****P < .0001)

To better understand the inflammatory condition of COVID-19 patients, we evaluated the expression levels of pro-inflammatory cytokines such as IL-6 and TNF-α transcripts. Considering that the patients had moderate to severe COVID-19, the expression levels of IL-6 and TNF-α were markedly increased compared to healthy controls (Fig. 3A-B).

### LncRNAs were highly correlated with important factors implicated in inflammation and fibrosis induction

**Fig. 4:**
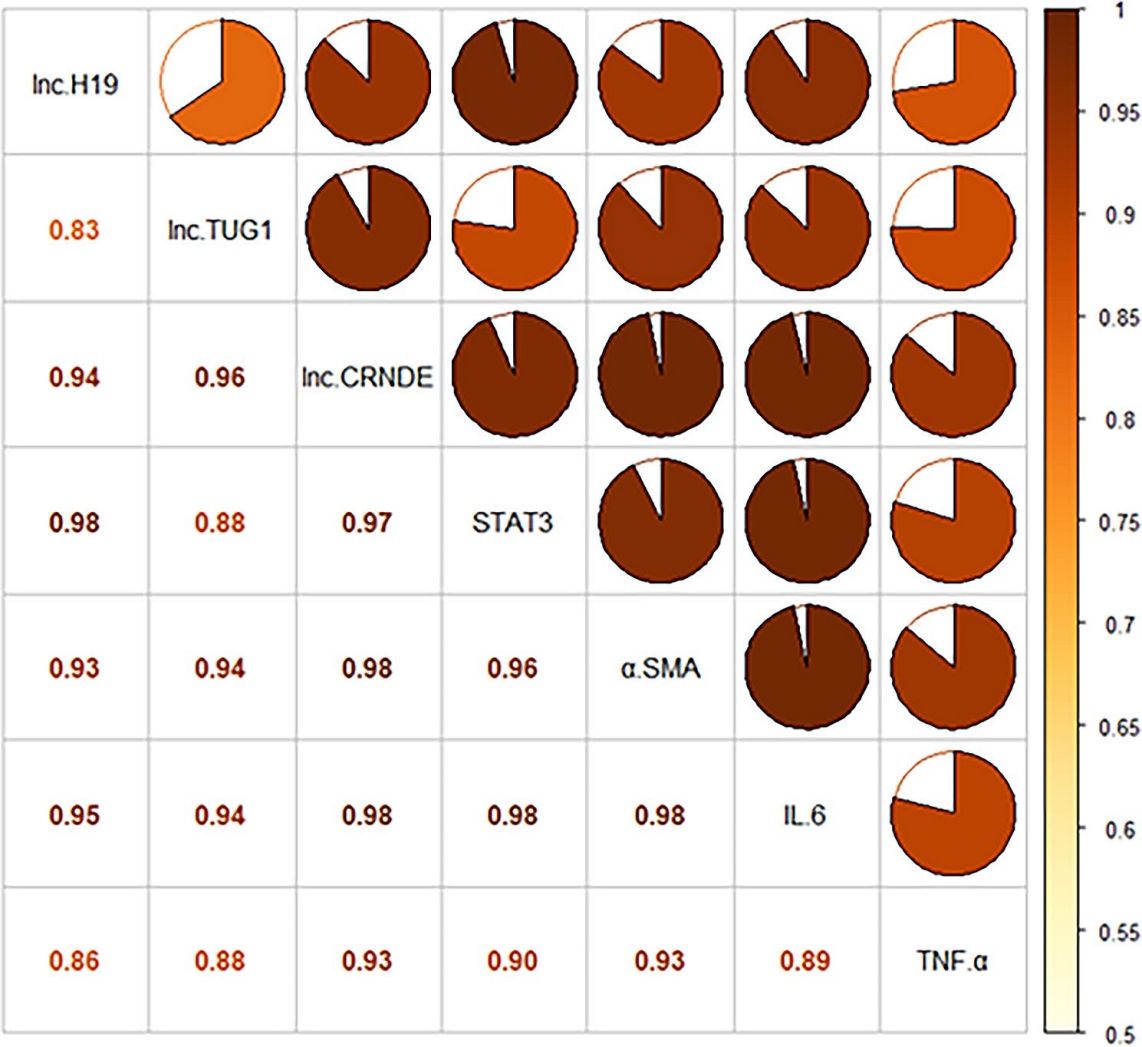
Correlation analysis. Correlation analysis was performed between three dysregulated lncRNAs (TUG1, CRNDE, and H19) in COVID-19 patients and four important genes involved in fibrosis in inflammation and induction (STAT3, α-SMA, TNF-α, and IL-6). The Pearson correlation coefficient for each pair was calculated using corrplot in R, revealing a high correlation with coefficients ranging from 0.83 to 0.98

We observed a significant elevation of three lncRNAs, namely TUG1, CRNDE, and H19, as well as four genes (STAT3, α-SMA, TNF-α, and IL-6) in COVID-19 patients compared to healthy controls in this study. Therefore, we conducted a correlation analysis to investigate their relationship. Utilizing the corrplot package in R, we calculated Pearson’s correlation coefficients, which demonstrated a high correlation among them, with correlation coefficients ranging from 0.83 to 0.98 (Fig. 4).

### Diagnostic biomarker panels

**Fig. 5:**
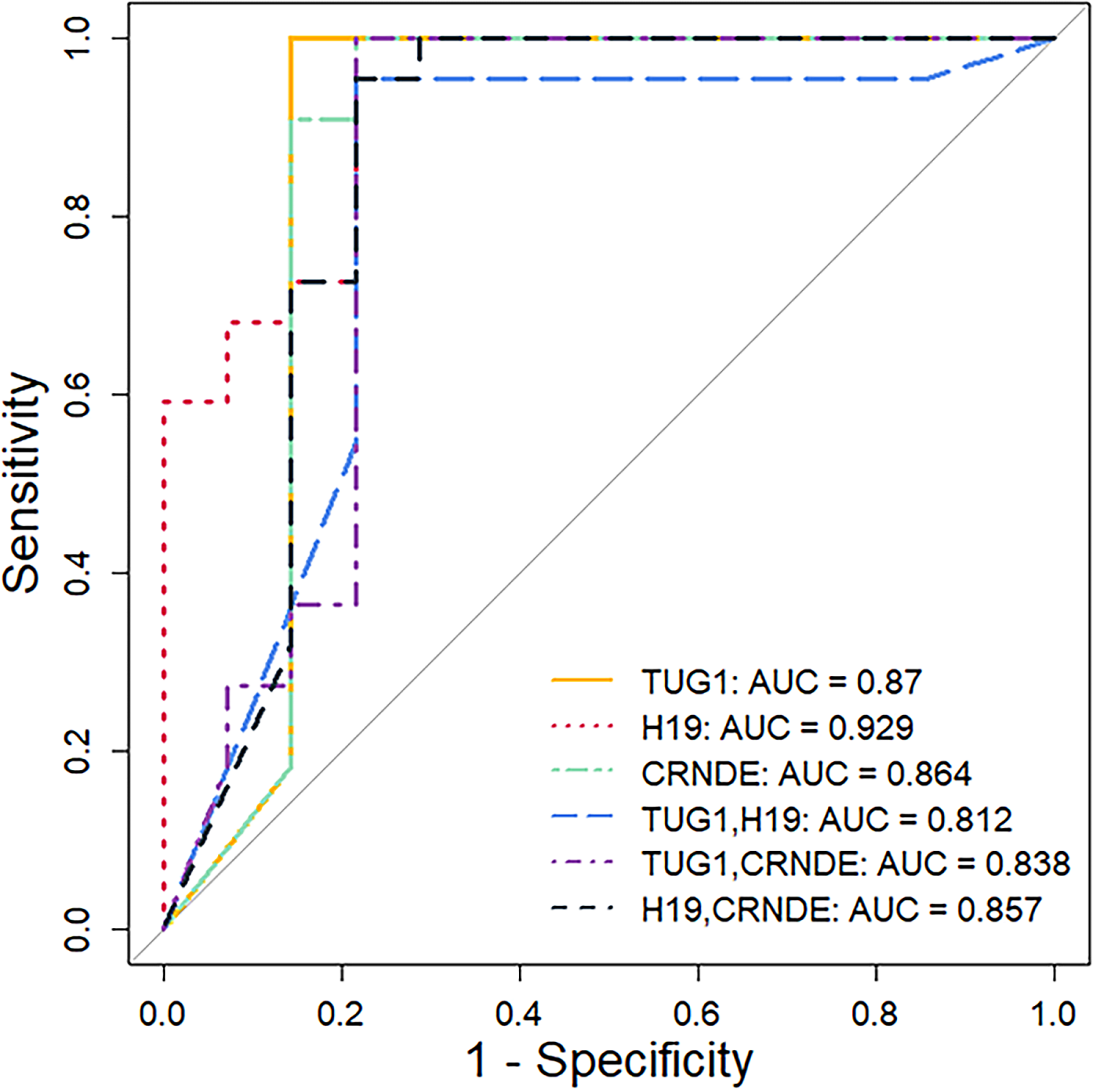
Evaluation of the diagnostic performance of selected lncRNAs in PBMCs of COVID-19 patients. ROC curve analysis of TUG1, H19, and CRNDE in distinguishing COVID-19 patients from healthy controls. The AUC showed the best diagnostic biomarker panel for patients with COVID-19

The expression levels of three lncRNAs H19, TUG1, and CRNDE demonstrated significant changes in COVID-19 patients compared to healthy individuals; hence, they were selected as inputs for the machine learning analysis. LR with five-fold cross-validation was employed to develop the predictive models, utilizing R programming packages. Within this cross-validation framework, 80% of the data was designated for training and the remaining 20% for testing, with this process iteratively repeated five times to reduce the risk of model overfitting. The performance of the models was assessed by reporting sensitivity, specificity, and accuracy, as shown in Table 4 Additionally, ROC curves for single and two-marker panels were presented in Fig. 5. lncRNA H19 displayed the highest performance among the single-marker panels, with an accuracy of 0.83 and an AUC of 0.929. For the two-marker panels, the combination of CRDNE and H19 yielded the best results, achieving an accuracy of 0.889 and an AUC of 0.857.

**Table 4:** The criteria of biomarkers to distinguish COVID-19 patients from control groups

Panel	Number of features	Sensitivity	Specificity	Accuracy
CRNDE	1	0.82	0.86	0.83
H19	1	0.86	0.79	0.83
TUG1	1	0.82	0.86	0.83
TUG1, CRNDE	2	0.86	0.79	0.83
H19, CRNDE	2	0.95	0.79	0.89
TUG1, H19	2	0.86	0.79	0.81

## Discussion

SARS-CoV-2 infection, like some other viruses, has the ability to dysregulate immune responses, especially dysregulation of T-cell lymphocytes. It can trigger a cytokine storm in the body and cause multiple immune responses, leading to malfunction in the corresponding organs. As mentioned, lncRNAs are among the important mediators of cellular responses and their expression could be influenced in various pathophysiological conditions like viral infections [43]. Herein, we attempted to investigate the effect of SARS-CoV-2 infection on three lncRNAs TUG1, H19, and CRNDE which were reported to be involved in many pathophysiological situations such as cancers [44], inflammatory states [45] and viral infections [21]. The expression level of lncRNA CRNDE has been reported to be increased in GC cells and tumors compared to control groups. Furthermore, Heydari et al. illustrated that lncRNA H19 was significantly upregulated in tissue and plasma EVs from active inflammatory bowel disease (IBD) patients compared to healthy individuals. LncRNA TUG1 has been also shown to be significantly overexpressed in COVID-19 patients compared to controls. The expression levels of TUG1 were positively correlated with the secretion of cytokines and chemokines, including CCL2 and TNF-α in plasma samples from COVID_19 patients [21]. Likewise, in this study, we showed remarkedly elevated expression levels of H19, TUG1, and CRNDE in PBMCs of COVID-19 patients with an AUC of 0.929, 0.87, and 0.864, respectively, indicating their ability to accurately differentiate the severe COVID-19 patients from the healthy controls in PBMC samples. Furthermore, we demonstrated that IL-6 and TNF-α levels were higher in PBMC specimens of moderate to severe COVID-19 patients than in healthy subjects and were correlated with three dysregulated lncRNAs. Khanaliha et al. proposed that the expression level of the lncRNAs H19 was significantly elevated in both severe and mild COVID-19 groups compared to the control group at the PBMC level, with an AUC of 0.76 for severe COVID-19 vs. mild COVID‐19, and 0.84 for mild COVID‐19 vs. control groups [46]. Although the role of these lncRNAs has been reported in the inflammatory phase of several diseases [45, 47, 48], the effects of their dysregulation on cellular processes in response to SARS-CoV-2 infection have not known and need further investigation. Dysregulation of H19 has also been shown to be associated with the onset of cardiometabolic diseases and has been proposed as a diagnostic marker and therapeutic target for these conditions [49]. TUG1, another overexpressed lncRNA observed in PBMCs of COVID-19 patients, may also be implicated in certain complications associated with COVID-19, such as inflammation and hypoxia, which are two key hallmarks of SARS-CoV-2 infection. Regarding the new role of TUG1 in the regulation of ROS production and aggravation of hypoxia-induced injury [50], Su et al. showed that TUG1sponges miR-132-3p and overexpresses HDAC3, leading to a decrease in H3K9 acetylation, inhibition of antioxidant genes expression, and enhancement of ROS production in the in vitro H2O2-stressed primary cardiomyocyte model [51]. Furthermore, we recently reported that the intracellular levels of ROS/O_2_^• −^ and expression levels of *CAT, NFE2L2, SOD1, SOD2* and *CYBB* genes, the five hub genes in the network constructed based on oxidative stress-related biological processes, protein-protein interactions, transcription factors, and co-expressed coefficients, were increased in PBMCs of COVID-19 patients [52]. Therefore, it seems that SARS-CoV-2 could disturb the redox equilibrium in the host cells by the overexpression of TUG1 as well as stress oxidative markers. Inflammatory cytokine production is very important in the exacerbation of COVID-19 symptoms as it may lead to multi-organ failure and death. In agreement with our findings, Tayel et al. demonstrated the TUG1 overexpression in the blood samples of moderate and severe COVID-19 patients with cytokine storm [21], thus TUG1 suppression could be a target in controlling the disease.

The last lncRNA, CRNDE, also exhibited an overexpressed pattern in COVID-19 patients compared to healthy controls. Depending on the disease context, it has been demonstrated that CRNDE activity could promote the cancer progression and viral infections [33, 35, 53]. One of the important signaling pathways affected by the overexpression of CRNDE is MyD88-independent toll-Like receptor (TLR)- signaling pathway wherein key factors including TLR3, TICAM1, PELI1, and RIPK2 are overexpressed. Intriguingly, several studies showed that TLRs, especially TLR3, could potentially be important in SARS-CoV-2 pathogenesis and interferon (IFN) response [54]. Therefore, there is compelling evidence indicating potential role of CRNDE in SARS-CoV-2 infection.

To gain further insight into how these lncRNAs might be involved in the induction of immune responses and disease pathogenesis, we analyzed the expression of STAT3 and α-SMA, a transcription factor and a fibrotic marker, respectively, in COVID-19 patients. lncRNA H19 has been shown to regulate non-small cell lung cancer (NSCLC) progression through STAT3 signaling by sponging miR-17 [41]. Furthermore, silencing of lncRNA TUG1 induced proliferation in TGF-β-treated BEAS-2B and HFL1 cells by inhibiting the expression levels of mesenchymal markers such as α-SMA and fibronectin [25].

STAT3 is a transcription factor that plays an important role in the immune cell proliferation, production of inflammatory cytokine, migration, and differentiation [55]. Indeed, STAT3 could be triggered by various cytokines, chemokines and growth factors in different physiological and pathological conditions [56–58]. Moreover, it has been reported that STAT3 could contribute to SARS-CoV-2 infection by decreasing the antiviral interferon response and triggering an imbalanced antiviral adaptive immune response. Considering its vital role in the dysregulation of inflammatory cytokines, it can be stated that STAT3 is a significant contributor in cytokine storm phenomenon observed in severe cases of COVID-19 [56, 59]. Furthermore, compelling evidence has revealed that lncRNAs can function as upstream regulators of STAT3 pathway. It has been discovered that TUG1 and H19 contribute to the overexpression of STAT3 in various cancers [55, 60, 61]. Consistent with these findings, we demonstrated a strong positive correlation between these lncRNAs (TUG1 and H19) and STAT3 in PBMCs of COVID-19 patients compared to healthy individuals.

Another important pathological characteristic of COVID-19 is pulmonary fibrosis [62, 63] in which α-SMA, a known marker of fibrosis is over-expressed [64]. Here, we reported a significant increase in α-SMA expression in PBMCs of COVID-19 patients, showing their vulnerability to lung fibrosis. The co-overexpression of CRNDE and α-SMA has been demonstrated in asthma-induced mice. Additionally, our study revealed a robust correlation between these two factors. Down regulation of CRNDE using siRNA decreased the expression of α-SMA and other EMT markers, leading to reduced lung inflammation and injury [42]. Likewise, the positive correlation between TUG1 with α-SMA, as demonstrated in this study, have been reported in the induction of hypoxia and cardiac fibrosis [65]. Yu et al. showed the profibrotic role of lncRNA H19 in precancerous oral submucous fibrosis. They found that H19 induced the overexpression of α-SMA, type I collagen, and fibronectin through the inhibition of miR-29b function [66]. These findings highlight the significant regulatory role of lncRNAs in COVID-19 pathogenesis and provide a valuable preliminary evaluation of lncRNAs as new targets for COVID-19 treatments.

Based on the differential expression of lncRNAs, a logistic regression approach was used to perform a classification analysis for diagnosing COVID-19 patients. We demonstrated that the expression of H19 and CRNDE in COVID-19 patients could accurately distinguish COVID-19 patients from healthy individuals with the AUC of 0.857. Given that dysregulation of lncRNAs has been previously reported in various pathophysiological conditions, the combination of these biomarker candidates could lead to achieve a more reliable diagnostic panel for COVID-19. In our previous studies, we showed that recognizing the host-specific biomarkers could be a promising complementary strategy to accurately diagnosing the SARS-CoV-2 infection from non-COVID cases [52, 67]. In addition, it has been shown that measuring the expression of lncRNAs could be useful in discriminating severe and moderate SARS-CoV-2 infections as well as monitoring the response of these patients to treatments [18, 68]. Thus, results of the models’ assessments indicate that lncRNAs could serve as a potential host-based biomarker for screening and precisely diagnosing COVID-19 patients from healthy controls.

## Conclusion

LncRNAs play important roles in the development and progression of various diseases. Our findings revealed that three lncRNAs that have been known to be important in many pathophysiological situations, were upregulated in PBMCs of moderate to severe COVID-19 patients. Furthermore, their co-expression with STAT3 and α-SMA, two important factors in the stimulation of inflammation and fibrosis, would highlight their possible contribution to exacerbation of symptoms and cardiovascular, lung, and prevalent complications of COVID-19. Furthermore, we introduced host-response-based diagnostic biomarker panels using machine learning algorithm, among which CRNDE-H19 panel indicated the greatest diagnostic power that could be applied as a complementary tool to diagnose the COVID-19 patients from non-COVID cases.

### Electronic supplementary material

Below is the link to the electronic supplementary material.

Supplementary Material 1

## Data Availability

The generated data during this study are included in the article. Further data are available from the corresponding author at a reasonable request.
