# Supplementary material for: Long non-coding RNAs in biomarking COVID-19: a machine learning-based approach
Source: Virol J. 2024 Jun 7;21:134. doi: 10.1186/s12985-024-02408-9 (PMC11161961; doi:10.1186/s12985-024-02408-9)
Supplement: Supplementary file 1 — Supplementary Material 1 [file 12985_2024_2408_MOESM1_ESM.docx]

**Long non-coding RNAs in biomarking COVID-19: a machine learning-based approach**

**Raheleh Heydari^1^, Mohammad javad Tavassolifar^1^, Sara Fayazzadeh^2^, Omid Sadatpour^3^, Anna Meyfour^1*^**

1. Basic and Molecular Epidemiology of Gastrointestinal Disorders Research Center, Research Institute for Gastroenterology and Liver Diseases, Shahid Beheshti University of Medical Sciences, Tehran, Iran
2. Bioinformatics and Computational Omics Lab (BioCOOL), Department of Biophysics, Faculty of Biological Sciences, Tarbiat Modares University, Tehran, Iran
3. Department of Immunology, School of Medicine, Tehran University of Medical Sciences, Tehran, Iran

*Address correspondence to: Anna Meyfour, Research Institute for Gastroenterology and Liver Diseases, Shahid Beheshti University of Medical Sciences, Arabi Ave., Daneshjoo Blvd., Velenjak, Tehran, Iran, Postal Code: 1985717413, Tel: +98 21 22432521 ([a.meyfour@gmail.com](mailto:a.meyfour@gmail.com)).

**A. Anti- α-SMA**

**STAT3**

50kDa


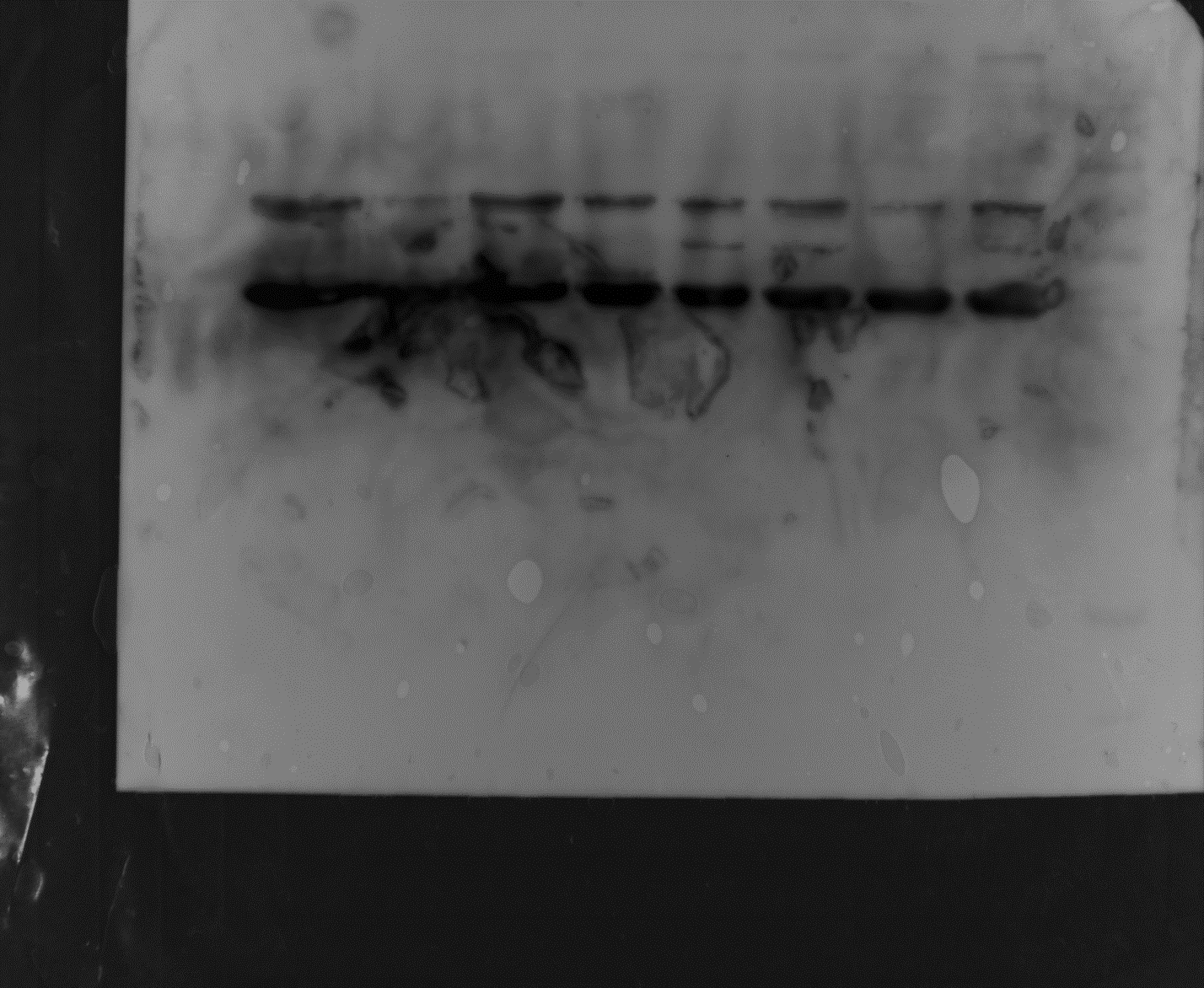


140 kDa

100 kDa

75 kDa

10 kDa

15 kDa

25 kDa

45 kDa

60 kDa

**B. Anti- STAT3**


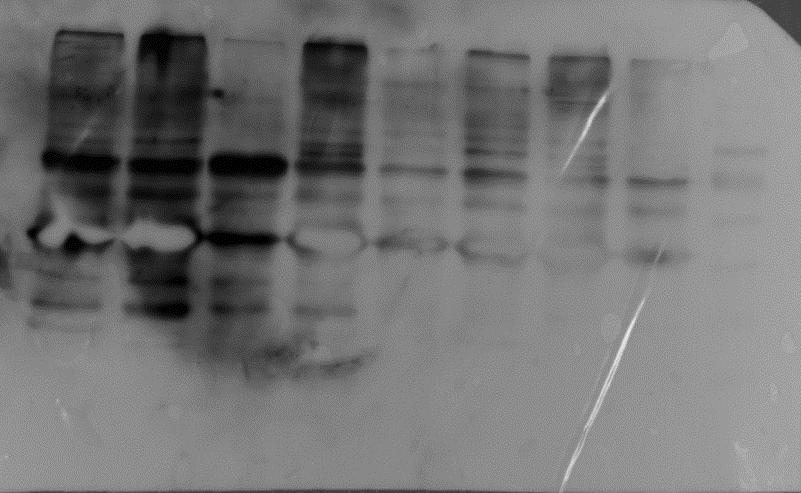


140 kDa

100 kDa

75 kDa

25 kDa

45 kDa

60 kDa

75 kDa

**C. Anti- ꞵ-Actin**

43 kDa


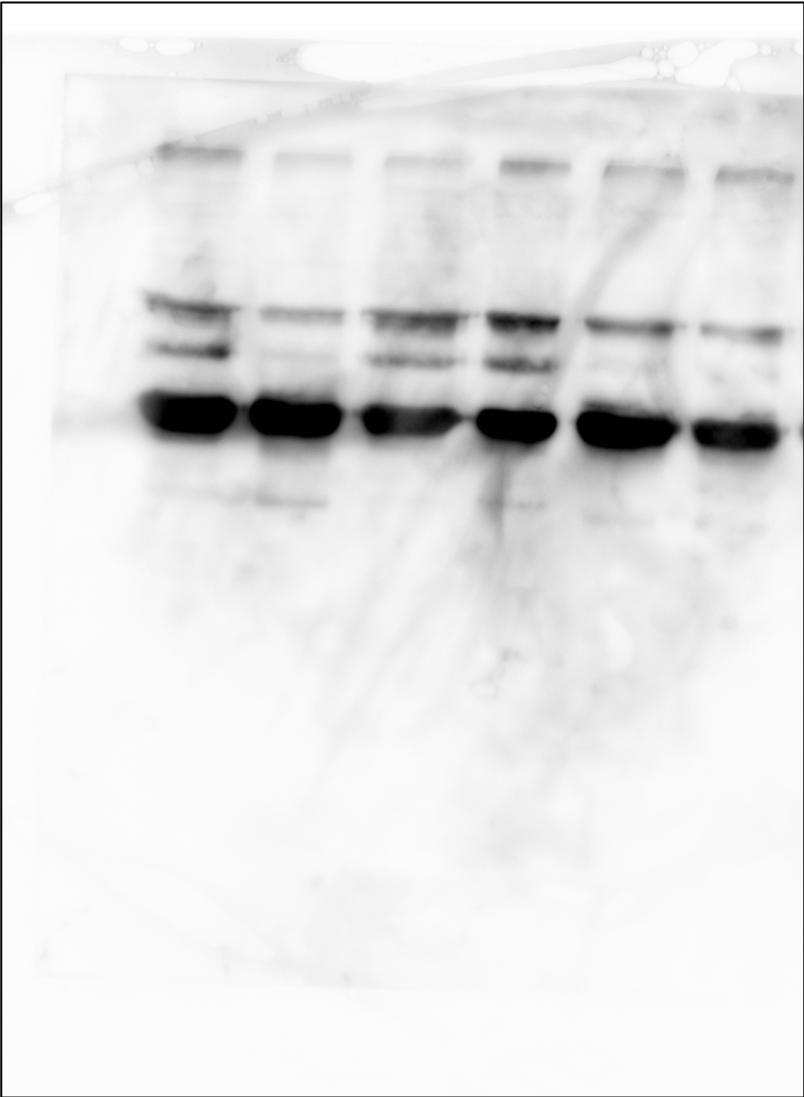


Supplementary Figure 1. Western blotting analysis to detect (A) α-SMA, (B) STAT3, and ꞵ-Actin (C) in COVID-19 patients and healthy controls.
